# Non-[^18^F]FDG PET-Radiopharmaceuticals in Oncology

**DOI:** 10.3390/ph17121641

**Published:** 2024-12-06

**Authors:** Antonia Dimitrakopoulou-Strauss, Leyun Pan, Christos Sachpekidis

**Affiliations:** Clinical Cooperation Unit Nuclear Medicine, German Cancer Research Center, Im Neuenheimer Feld 280, 69120 Heidelberg, Germany

**Keywords:** PET radiopharmaceuticals, oncology, immuno-PET, total-body PET

## Abstract

Molecular imaging is a growing field, driven by technological advances, such as the improvement of PET-CT scanners through the introduction of digital detectors and scanners with an extended field of view, resulting in much higher sensitivity and a variety of new specific radiopharmaceuticals that allow the visualization of specific molecular pathways and even theragnostic approaches. In oncology, the development of dedicated tracers is crucial for personalized therapeutic approaches. Novel peptides allow the visualization of many different targets, such as PD-1 and PD-L1 expression, chemokine expression, HER expression, T-cell imaging, microenvironmental imaging, such as FAP imaging, and many more. In this article, we review recent advances in the development of non-[^18^F]FDG PET radiopharmaceuticals and their current clinical applications in oncology, as well as some future aspects.

## 1. Introduction

Positron emission tomography (PET) in combination with computed tomography (CT) is the most widely used molecular imaging modality for diagnosis and therapy monitoring, particularly in oncology. The recently developed long-axial field-of-view (LAFOV) and total-body PET-CT systems offer much higher sensitivity (up to 40 times higher) than conventional standard-axial field-of-view (SAFOV) scanners and therefore increase lesion detectability by delineating very small lesions. They also enable low-dose imaging, delayed scanning, and the reduction in the applied dose of a radiotracer, as well as shorter scanning protocols [1]. All these advantages make its use within personalized treatment approaches more attractive. PET-CT with dedicated radiopharmaceuticals can help to tailor oncological therapy by identifying non-responders early in the course of treatment, changing a therapeutic protocol to a more effective one, escalating the dose, or discontinuing therapy in the case of complete metabolic remission, especially with new immunotherapies, to avoid unnecessary side effects and costs [2,3].

The most commonly used radiopharmaceutical in oncological PET-CT is ^18^F-fluorodeoxyglucose ([^18^F]FDG), a metabolically active tracer that is transported by several glucose transporters and phosphorylated by various hexokinases and then trapped. [^18^F]FDG is enhanced in most malignancies and is widely available [4]. Despite its high sensitivity, it lacks specificity due to some non-specific uptake in inflammatory lesions, post-operative changes, post-radiation changes, etc.

It is known that, despite decades and billions of dollars spent on research, less than 1% of the radiotracers developed reach the clinic [5]. One reason for this may be the lack of knowledge about the full range for the entirety of molecules that can be targeted and used as radiopharmaceuticals, as well as their clinical relevance [6]. The aim of this review is to provide an overview of some known and, in particular, novel non-[^18^F]FDG radiopharmaceuticals for oncological applications.

## 2. Metabolically Active Tracers Beyond [^18^F]FDG: Proliferation Tracers, Labeled Amino Acids, Lipid Metabolism

**Figure 1:**
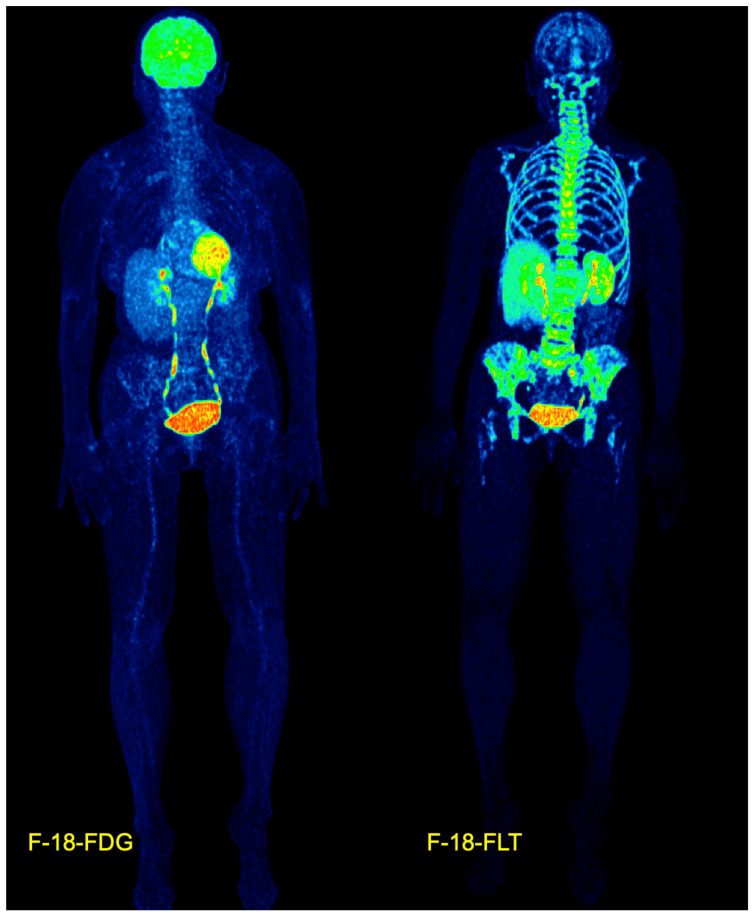
Maximum intensity projection (MIP) images of a patient with multiple myeloma 1 h after i.v. [^18^F]FDG injection (left side) and 1 h after [^18^F]FLT injection (right side). The [^18^F]FLT images show high uptake in the bone marrow of the axial skeleton, the pelvic bones, the spleen, and the liver. The [^18^F]FDG images show no focal or diffuse enhanced uptake in the bone marrow. Normal [^18^F]FDG excretion in the urinary tract.

We know that [^18^F]FDG is related to cell viability and correlates with many tumor characteristics, such as proliferation, hypoxia, angiogenesis, apoptosis, etc. [7,8,9,10,11,12]. Attempts have been undertaken to develop dedicated tracers for tumor cell proliferation. The most promising tracer is ^18^F-fluorodeoxythymidine ([^18^F]FLT), which is transported and phosphorylated but not incorporated into DNA after phosphorylation [13]. However, [^18^F]FLT reflects the rate of DNA replication and is thought to be more specific than [^18^F]FDG because it is not taken up by cells involved in an inflammatory process. [^18^F]FLT has some limitations and has not found the same clinical use as [^18^F]FDG. One reason is the high [^18^F]FLT uptake in normal bone marrow and liver, which is an obstacle for the assessment of bone diseases, including hematological neoplasms such as multiple myeloma and leukemia, as well as for the assessment of liver metastases [14,15,16] (Figure 1). Another limitation is that [^18^F]FLT uptake is often lower than that of [^18^F]FDG; therefore, its sensitivity is lower than that of [^18^F]F-FDG, but its specificity may be higher [15]. False positive results have also been reported with [^18^F]FLT; e.g., this occurred due to pseudoprogression two weeks after anti-PD-1 therapy in 26 patients with advanced non-small cell lung cancer (NSCLC) [17]. Overall, the number of patient studies published to date is significantly lower than with [^18^F]FDG, and it is therefore unclear whether the widespread use of [^18^F]FLT is limited by insufficient availability or by the above-mentioned limitations. Carbon-11-labeled thymidine has been developed but is not widely used because of the short half-life of 20 min and the rapid catabolism of thymidine. Other proliferation tracers, such as [^18^F]FMAU or [^18^F]F-AraG, have found only limited use [18].

Tumor cells demonstrate enhanced amino acid transport due to increased protein synthesis. Therefore, several amino acids have been labeled over the years, mostly with carbon-11. Well-known tracers are [^11^C]methionine, [^11^C]glutamate, [^11^C]choline, and [^11^C]acetate. ^18^F-labeled amino acids have been developed to overcome the problem of the short half-life of carbon-11. The most commonly used ^18^F-labeled amino acids are [^18^F]FET, and [^18^F]F-DOPA. [^18^F]FET is widely used in patients with gliomas due to the high specific uptake in the tumor and the lack of uptake in the normal brain parenchyma. An overview of the use of PET-labeled amino acids in neuro-oncology is provided by Galldiks et al. [19]. [^18^F]F-DOPA is used to diagnose Parkinson’s disease and in oncology to diagnose neuroendocrine tumors. However, [^18^F]F-DOPA is no longer the tracer of choice for neuroendocrine tumors following the development of labeled somatostatin analogues that can be used not only for diagnostic purposes but also for theragnostic approaches.

Both ^11^C- and ^18^F- labeled choline have been developed for imaging lipid metabolism. In particular, [^18^F]fluorocholine has been the tracer of choice for the detection of prostate cancer, but it is also commonly applied for other malignancies, such as multiple myeloma [20]. In the last decade, [^18^F]fluorocholine has ceased to be the tracer of choice for prostate cancer because specific peptides have been developed, such as various prostate-specific membrane antigen (PSMA) ligands, which are much more sensitive and specific than labeled choline. The newly developed PSMA ligands are small peptide mimetics that are rapidly cleared from the circulation, resulting in low background activity early after injection, and they have already found clinical use in theragnostic applications.

## 3. Hypoxia Tracers

Hypoxia is an important feature of solid tumors and has particular implications for the impact of radiation treatment planning because hypo-oxygenation can lead to resistance to radiotherapy through several mechanisms. One is that the lack of oxygen prevents the formation of reactive oxygen, which can lead to permanent DNA damage; another is the expression of hypoxia-inducible factors (HIFs), which lead to other resistance mechanisms [21]. The most commonly used hypoxia PET tracers are [^18^F]F-misonidazole ([^18^F]FMISO), [^18^F]FAZA, [^18^F]F-HX4, and [^64^Cu]Cu-ATSM. Details about these tracers are provided elsewhere [18].

## 4. Targets for Tumor Microenvironment (TME)

Most PET radiopharmaceuticals used in the past targeted more or less different metabolic pathways of the tumor cells. The extraordinary advances in immunotherapy have opened a new era for tracers which target the TME. In addition to cancer cells, the TME includes, cancer-associated fibroblasts (CAFs), endothelial cells, myeloid-derived suppressor cells (MDSCs), tumor-infiltrating lymphocytes (TILs), the extracellular matrix, and vasculature [22,23].

A novel approach targeting the TME is the use of fibroblast activation protein inhibitor (FAPI) imaging in oncological patients, as FAP is known to be overexpressed in the cancer-associated fibroblasts (CAFs) of many solid tumors. Currently, several FAPI analogues are available, labeled with ^68^Ga or ^18^F, such as [^68^Ga]Ga-FAPI-46 and [^18^F]F-FAPI-74. The initial results demonstrate that FAPI may be superior in tumors with low [^18^F]FDG uptake [24,25]. In a meta-analysis published by Sollini et al., the pooled sensitivity was 0.99 (95% CI 0.97–1.00), and the pooled specificity was 0.87 (95% CI 0.62–1.00) in a patient-based analysis with negligible heterogeneity [26]. In the same paper, the authors reported on a high heterogeneity in a lesion-based analysis. The pooled sensitivity was 1.00 (95% CI 0.98–1.00) for primary tumors and 0.93 (95% CI 0.88–0.97) for distant metastases. The results for the detection of nodal metastases were heterogeneous and worse than for primary tumors and distant metastases. The conclusion of this paper was that FAPI has potential but does not appear to be able to replace FDG at this stage. The studies published to date have methodological drawbacks, such as a limited number of patients, retrospective design, heterogeneous patient cohorts with a mix of different tumor entities and indications, including primary diagnosis, restaging, radiation treatment planning, and therapy monitoring [26].

## 5. Novel Peptides for Theragnostic Applications: SSTR2, PSMA, FAPI

**Figure 2:**
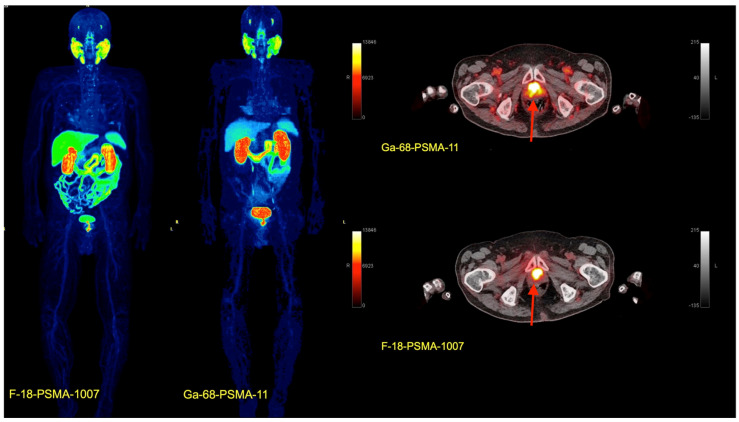
Example of a patient with biochemical recurrence of prostate cancer. MIP images 1.5 h after i.v. injection of [^18^F]PSMA-1007 (left side) and 1.5 h after application of [^68^Ga]Ga-PSMA-11 (right side) on the following day due to some non-specific bone uptake of [^18^F]PSMA-1007. Fused transversal images of a prostate cancer recurrence (red arrow) with both tracers. Of note is the different distribution of the two tracers. Enhanced hepatobiliary excretion of the [^18^F]PSMA-1007. Increased urinary excretion of [^68^Ga]Ga-PSMA-11.

Receptor-active peptides enable both the diagnosis of and the therapy for receptor-active tumors and are used in theragnostic approaches. One of the first examples was [^68^Ga]Ga-DOTA-TOC, a tracer that binds to somatostatin receptor 2 (SSTR2)-expressing tumors, such as neuroendocrine carcinomas or meningiomas, and it is also used for therapy in DOTA-TOC-positive tumors labeled with beta emitters as ^90^Y or ^177^Lu. The selection of suitable patients and the positioning of peptide receptor radionuclide therapy (PRRT) with SSTR receptor ligands are based on different criteria, such as SSTR expression in PET-CT images, but also on [^18^F]FDG-uptake, the proliferation rate based on the ki67 index, and other aspects, which are discussed in a review by Albertelli et al. [27]. Another example of PRRT is PSMA (prostate-specific membrane antigen) compounds, which bind to PSMA, a cell surface protein, that is enhanced in prostate carcinoma (Figure 2). The first PSMA used for diagnosis was [^68^Ga]Ga-PSMA-11, followed by [^18^F]PSMA-1007. The introduction of these new tracers improved the detection of tumor recurrence and the staging of prostate cancer by detecting unknown metastatic lesions with a high contrast to the surrounding tissue [28,29]. PSMA ligands labeled with either ^90^Y or ^177^Lu, most commonly [^177^Lu]Lu-PSMA-617, are used for PRRT in PSMA-avid tumors mostly as last-line therapy, but not exclusively [30]. A review of the different PSMA ligands as well as the pros and cons for both diagnosis and theragnostics is provided elsewhere [31].

The results of PRRT with FAPI ligands are less promising compared to DOTA-TOC and PSMA. In a review of FAPI theragnostics, Haberkorn et al. provided an explanation for the limited success of these ligands. They mentioned that the main problem is the heterogeneity of the FAP expressed in the stromal cells and that only a few tumors, such as sarcomas and mesotheliomas, have FAP-positive tumor cells. The authors suggest the use of heterodimers such as FAPI-RGD for imaging purposes [32].

## 6. Tracers for Immuno-PET

### PD-1 and PDL-1 Imaging, T-Cell Imaging

Immune checkpoint inhibitors (ICIs) have revolutionized cancer therapy by activating the patient’s immune system by blocking T-cell inactivation. The first ICI was ipilimumab, a cytotoxic T-lymphocyte-associated protein 4 (CTLA-4) inhibitor, followed by inhibitors of programmed cell death protein 1 (PD-1) and programmed death-ligand 1 (PD-L1) as monotherapy or in combination regimens, either as a combination of different ICIs or in combination with chemotherapy [33]. Dedicated radiopharmaceuticals for immunoimaging have been in development for several years and have been used mainly in preclinical studies, but also in a limited number of clinical trials. Various targets have been labeled for this purpose, most of which target PD-1 and PD-L1 expression for non-invasive whole-body assessment.

The first studies in patients with NSCLC and the tracers [^89^Zr]Zr-nivolumab, [^89^Zr]Zr-atezolizumab, or [^18^F]F-BMS-986192 have been published [34,35]. The authors demonstrated a significant heterogeneity of these tracers between patients as well as within different tumor lesions of the same patient. Verhoeff et al. used [^89^Zr]Zr-DFP-durvalumab, an anti-PD-L1 ligand, prior to durvalumab monotherapy in 33 patients with recurrent or metastatic head and neck cancer in a prospective multicenter phase I-II study. They could demonstrate the feasibility and safety of [^89^Zr]Zr-DFO-durvalumab PET/CT, but the [^89^Zr]Zr-DFO-durvalumab uptake did not correlate with the durvalumab response [36]. Zhang et al. developed four PD-L1-specific tracers ([^68^Ga]Ga-NOTA-RW102, [^68^Ga]Ga-NOTA-ABDRW102, [^64^Cu]Cu-NOTA-ABDRW102, and [^89^Zr]Zr-DFO-ABDRW102) with different circulation times and evaluated them in preclinical PD-L1-positive solid tumor models as well as in patients (n = 10) with NSCLC. They concluded that immuno-PET imaging with [^68^Ga]Ga-NOTA-RW102 holds promise for visualizing differential PD-L1 expression, selecting patients for PD-L1-targeted immunotherapies, and monitoring immune-related adverse effects in patients receiving PD-L1-targeted treatments [37]. Preclinical results exist on labeled nanobodies (Nbs) and affibodies as targets for PD-L1 imaging. Bridoux et al. validated an anti-human PD-L1 nanobody (NB), [^68^Ga]Ga-NOTA-(hPD-L1) Nbs, with two different labeling techniques and demonstrated stability and excellent in vivo targeting with >95% radiochemical purity [38]. González Trotter et al. radiolabeled the PD-L1-binding affibody molecule NOTA-ZPD-L1_1 with ^18^F and evaluated its in vitro and in vivo binding affinity and targeting, as well as its specificity in tumor-bearing mice with different PD-L1 expressions. PET imaging demonstrated rapid tracer uptake in the PD-L1-positive tumor, whereas the PD-L1-negative control tumor showed little tracer retention; the conclusion was that affibody ligands can be effective for in vivo PD-L1 imaging [39]. Another promising immune checkpoint molecule is lymphocyte activation gene-3 (LAG3). LAG-3 inhibitors are used in some cancers, such as head and neck cancer, lung cancer, and metastatic melanoma. Miedema et al. labeled an anti-LAG-3 antibody with ^89^Zr and investigated the potential use of [^89^Zr]Zr-BI 754111 in a limited number of patients with head and neck cancer (n = 2) and with lung cancer (n = 4). They used the pre-administration of unlabeled BI 754111 to investigate target specificity. They concluded that this tracer has the potential to be used as a predictive imaging biomarker for LAG-3-targeted therapies [40].

Another very interesting approach is the so-called T-cell imaging. The first results have recently been published using an [^89^Zr]Zr-anti-CD8 minibody ([^89^Zr]Zr-IAB22M2C) in six patients with solid tumors, including melanoma, lung tumors, and hepatocellular carcinoma. The authors observed the best detectability of tumor lesions 24 h p.i. [41]. In a breast cancer model, Lu et al. used [^89^Zr]Zr-DFO-CD4 and [^89^Zr]Zr-DFO-CD8 as biomarkers to assess response to ICI therapy. They concluded that imaging metrics of CD8+ and CD4+ cells may predict the response to immunotherapy and guide clinical decision making [42].

## 7. Human Epidermal Growth Factor Receptor, Estrogen Receptor Imaging

Human epidermal growth factor receptor (HER) imaging has also been used in preclinical studies and in a limited number of patients. The HER proteins are among the most studied receptor tyrosine kinases and consist of four HER proteins, namely HER1 (also known as EGFR), HER2, HER3, and HER4. Mohr et al. studied five patients with metastatic breast cancer with [^89^Zr]Zr-trastuzumab, a monoclonal antibody used to treat HER2 receptor-positive tumors. HER2 imaging may help select patients prior to treatment [43]. Yeh et al. studied six patients with HER2-positive metastatic breast cancer with [^89^Zr]Zr-pertuzumab to assess HER2 status and heterogeneity to guide biopsy and decide the next line of treatment at progression. The tracer detected known sites of malignancy, indicating that these tumor lesions were HER2-positive. The optimal imaging time point was 5–8 days after administration, and no toxicities were observed. The authors suggest further potential applications such as real-time evaluation for biopsy guidance [44]. Menke-van der Houven van Oordt et al. used [^89^Zr]Zr-anti-HER3 mAb (GSK2849330) to monitor six patients with HER3-positive solid tumors. Furthermore, they used dose-dependent tracer inhibition by increasing doses of unlabeled GSK2849330 in combination with the tracer. The tracer imaging showed good tumor uptake in all the evaluable patients. Pre-dosing with unlabeled mAb reduced the tumor uptake rate in a dose-dependent manner. The authors concluded that this approach not only allows visualization of HER receptors but also offers potential for dose selection [45]. [^89^Zr]Zr-cetuximab is another HER1-targeting tracer that was used in nine patients (six with NSCLC and three with head and neck tumors) within a phase 1 study by van Loon et al. The authors found increased tracer uptake in eight/nine patients with a tumor-to-blood ratio > 1 and recommended late scanning at least 6 days p.i. [46]. [^89^Zr]Zr-lumretuzumab, which targets anti-HER3, was applied in 20 patients with HER3-expressing solid tumors prior and after lumretuzumab treatment [47]. The results showed high uptake in 67.6% of CT-delineated tumor lesions with a diameter of at least 1 cm. In a patient-based analysis, 19/20 patients had at least one tumor lesion with enhanced uptake. The authors investigated tumor saturation with different doses of cold lumretuzumab but found no clear evidence of tumor saturation by PET, as tumor SUV did not reach a plateau with increasing doses [47].

Estrogen receptors are overexpressed in approximately 70% of all breast cancers and can be visualized by [^18^F]-fluoro-17β-estradiol (FES). A review on this topic is presented by van Kruchten et al. [48]. The authors state that measurements of tumor protein expression in estrogen receptors as measured by [^18^F]FES detected estrogen receptor-positive tumor lesions with a sensitivity of 84% and specificity of 98% as compared with biopsy. Furthermore, 45% of patients with metastatic breast cancer have discordant estrogen receptor expression across lesions.

## 8. Chemokine Receptor Imaging

Chemokine receptor 4 (CXCR4) is overexpressed in various solid cancers and has been used as a target for molecular imaging using [^68^Ga]Ga-pentixafor. Dreher et al. studied 142 patients with 23 different histologically proven solid tumors and could demonstrate that 103/152 (67.8%) scans showed detectable uptake above the blood pool (TBR  >  1) in 462 lesions (52 primary tumors and 410 metastases). The median TBR was 4.4, indicating high image contrast. The highest SUVmax was observed in ovarian cancer, followed by small cell lung cancer, a desmoplastic small round cell tumor, and adrenocortical carcinoma. Interestingly, a fraction of the patients with [^68^Ga]Ga-pentixafor-positive tumor lesions were potentially suitable for CXCR4-directed therapy. Furthermore, they observed a weak but statistically significant correlation between in vivo and ex vivo CXCR4 expression, suggesting high specificity of the PET agent [49]. [^68^Ga]Ga-pentixafor has also been used in hematological malignancies, such as multiple myeloma and lymphoma [50,51]. Buck et al. studied 690 different solid and hematologic malignancies with the same tracer and demonstrated high image contrast in a variety of neoplasms, particularly in hematological malignancies, small cell lung cancer, and adrenocortical neoplasms. Multiple myeloma showed the highest uptake with an SUVmax > 12 [52]. Theragnostic approaches with ^177^Lu- or ^90^Y-labelled pentixafor in a limited number of patients have been published, mainly those with hematologic malignancies [53].

## 9. Vascular Endothelial Growth Factor A Imaging

Vascular endothelial growth factor A (VEGF A) is overexpressed in several malignant lesions and has found limited use as [^89^Zr]Zr-bevacizumab or [^95^Nb]Nb-bevacizumab as well as [^52^Mn]Mn-DOTAGA-bevacizumab, mostly in preclinical studies [54,55,56]. Gaymeka et al. studied 23 breast cancer patients with 26 breast tumor lesions with [^89^Zr]Zr-bevacizumab and demonstrated an enhanced uptake in 25/26 breast tumors. The only lesion not detected was an invasive ductal carcinoma with a diameter of 10 mm. In addition, 4/10 axillary regions with at least one lymph node metastasis were positive in PET. No false positives were reported in this study [57]. Oosting et al. studied 26 patients with metastatic renal cell carcinoma (mRCC) with [^89^Zr]Zr-bevacizumab before and after antiangiogenic therapy with either bevacizumab with interferon or sunitinib and compared the uptake with plasma VEGF-A levels and time to disease progression [58]. They reported a high [^89^Zr]Zr-bevacizumab uptake in mRCC with remarkable inter- and intra-patient heterogeneity. The reduction in uptake was greater after bevacizumab with interferon therapy and modest after sunitinib therapy. High baseline uptake was associated with longer time to progression, but tumor uptake was not related to plasma VEGF-A in any scan [58]. [^89^Zr]Zr-bevacizumab was also used in a similar design in 14 patients with advanced well-differentiated neuroendocrine tumors prior and after everolimus therapy, which is an mTOR inhibitor with multiple antitumor effects, including the reduction in angiogenesis [59]. The results showed variable tumor uptake prior to therapy and a decrease after therapy. In 10/14 patients, 63 tumor lesions were delineated, representing 19% of the lesions of at least 1 cm in CT. The authors concluded that [^89^Zr]Zr-bevacizumab imaging might be useful as an early predictive biomarker of anti-VEGF-directed treatment [59]. The same tracer was applied in seven patients with advanced NSCLC and showed high tumor uptake [60].

## 10. Other Targets

The chimeric monoclonal antibody anti-CD44 [^89^Zr]Zr-U36 was used in 20 patients with squamous cell carcinoma of the head and neck who were at high risk of neck lymph node metastases. The tracer detected all primary tumors and lymph node metastases in 18/25 positive levels. The authors concluded that [^89^Zr]Zr-U36 performed at least as well as CT/MRI for the detection of lymph node metastases [61].

CD20 and CD38 antigens are other targets that have been used not only preclinically but also in a few patients with hematologic malignancies. Muylle et al. used [^89^Zr]Zr-rituxumab, a chimeric IgG1 kappa monoclonal antibody targeting CD20, in five patients with CD20+ B-cell lymphoma and studied the impact of preloading with unlabeled rituximab on tumor targeting and a radiation dose of [^90^Y]Y-rituximab therapy. The authors showed that administration of the standard preload of unlabeled rituximab impairs tumor targeting of the radioconjugate in the majority of patients eligible for radioimmunotherapy and suggest that this common practice should be reconsidered [62]. CD-38-targeted immuno-PET was used with [^89^Zr]Zr-DFO-daratumumab in 10 patients with multiple myeloma in a phase 1 study and demonstrated high uptake in osseous deposits; it was concluded that this tracer may be useful not only for diagnosis but also for the evaluation of minimal residual disease and the prediction of daratumumab therapy [63].

Another interesting target is mesothelin (MSLN), which is overexpressed in several solid tumors. Lamberts et al. used [^89^Zr]Zr-MMOT0530A, an anti-MSLN antibody, in 11 cancer patients (7 with pancreatic cancer and 4 with ovarian cancer) and observed enhanced tumor uptake in at least one tumor lesion in all the patients, as well as heterogeneity in tumor uptake between and within patients [64].

Trop2, also known as trophoblast cell surface antigen 2, is a tumor-associated calcium signal transducer (TACSTD) gene family member and another interesting target. Trop2 facilitates tumor development by mediating multiple signaling pathways and promoting tumor cell growth, proliferation, and metastasis [65]. Huang et al. developed two ^68^Ga-labelled nanobody tracers that target Trop2 [66]. [^68^Ga]Ga-NOTA-T4 demonstrated excellent pharmacokinetics in preclinical solid tumor models and ten patients with solid tumors. [^68^Ga]Ga-NOTA-T4 immuno-PET could facilitate clinical decision making through patient stratification and response monitoring during Trop2-targeted therapies [66].

**Figure 3:**
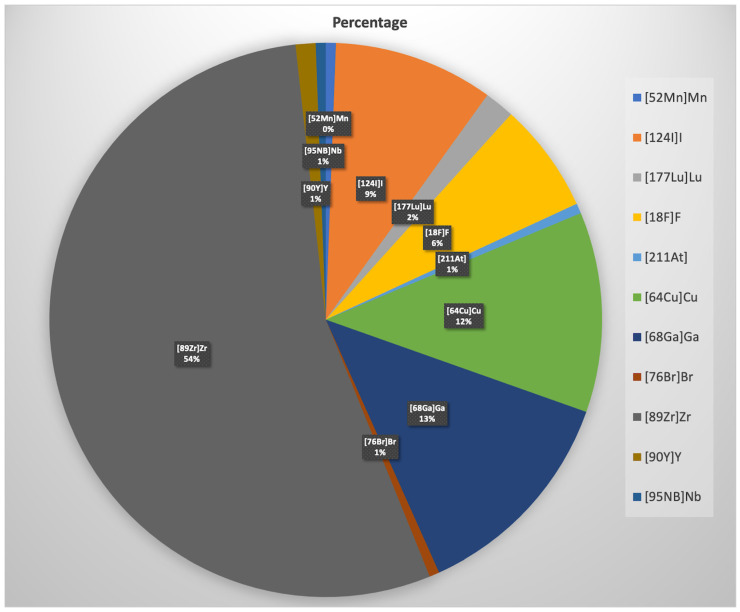
Pie chart showing the percentage of radionuclides used for labeling of different targets for PET immunoimaging.

A summary of the tracers used for immuno-PET discussed in this paper is presented in Table 1. Many other targets have been labeled and investigated in a preclinical setting, such as CD105, CD133, CD166, CD192, CD44, glypican, CEA, claudin, mucin, RANKL, and TNF. A nice overview of the target selection, developability, translatability, and diagnostic quality of different immunoimaging biomarkers can be found elsewhere [67]. The majority (54%) of these novel targets have been labeled with ^89^Zr, as demonstrated in Figure 3. A systematic review of human applications of ^89^Zr-labeled radiopharmaceuticals in oncological and non-oncological applications is provided by De Feo et al. [68]. Attempts are undertaken to develop pipelines based on deep learning-based hybrid human–AI pipelines by bridging individual genes and their relevance in human diseases with specific molecular imaging methods in order to accelerate the transition from the molecular level to the clinical translation [6].

## 11. Conclusions

To date, a variety of non-FDG radiopharmaceuticals have been used in oncology for primary and recurrent diagnosis, staging, therapy monitoring, and molecular characterization of a tumor and its metastases (Table 2). These include:

- Proliferation tracers like [^18^F]FLT and [^18^F]FMAU;
- Labeled amino acids like [^11^C]methionine, [^11^C]glutamate, [^18^F]FET, and [^18^F]FDOPA;
- Tracers for lipid metabolism like [^11^C]- and [^18^F]-labeled choline and [^11^C]acetate;
- Hypoxia tracers like [^18^F]FMISO, [^18^F]-FAZA, [^18^F]F-HX4, and [^64^Cu]Cu-ATSM;
- Targets for the microenvironment like [^68^Ga]Ga-FAPI-46, [^18^F]F-FAPI-74, [^90^Y], and [^177^Lu]-FAPI;
- Receptor-active peptides for theragnostic applications like [^68^Ga]Ga-DOTA-TOC, [^90^Y]- or [^177^Lu]-DOTA-TOC, [^68^Ga]Ga-PSMA-11, [^18^F]-PSMA-1007, [^177^Lu]-PSMA-617, [^68^Ga]Ga-pentixafor, and [^90^Y]-pentixafor;
- PD-1 and PDL-1 imaging with tracers like [^89^Zr]Zr-nivolumab, [^89^Zr]Zr-atezolizumab, [^18^F]F-BMS-986192, [^89^Zr]Zr-DFP-durvalumab, and [^68^Ga]Ga-NOTA-RW102;
- LAG-3 imaging with tracers like [^89^Zr]Zr-BI 754111;
- T-cell imaging with tracers like [^89^Zr]Zr-anti-CD8 minibody;
- HER imaging with tracers like [^89^Zr]Zr-trastuzumab and [^89^Zr]Zr-cetuximab;
- Estrogen receptor imaging with tracers like [^18^F]FES;
- VEGF A imaging with tracers like [^89^Zr]Zr-bevacizumab;
- [^89^Zr]Zr-U36 (anti-CD44);
- [^89^Zr]Zr-rituxumab and [^90^Y]Y-rituximab (anti-CD20) for theragnostic applications;
- [^89^Zr]Zr-DFO-daratumumab (anti-CD38);
- [^89^Zr]Zr-MMOT0530A (mesothelin);
- [^68^Ga]Ga-NOTA-T4 (Trop2 imaging);
- Other targets for preclinical research: CD105, CD133, CD166, CD192, CD44, glypican, CEA, claudin, mucin, RANKL, and TNF.

## Figures and Tables

**Table 1:** A summary of PET tracers used for immuno-PET.

Tracer	Target	Reference	Tumor Type	No. of Patients
[^89^Zr]Zr-nivolumab, [^18^F]F-BMS-986192	PD-1	34	NSCLC	13 patients with NSCLC with [^89^Zr]Zr-Nivolumab and [^18^F]F-BMS
[^89^Zr]Zr-atezolizumab	PD-L1	35	NSCLC, bladder cancer, breast cancer	25 patients with locally advanced or metastatic bladder cancer, NSCLC, triple-negative breast cancer
[^18^F]F-BMS-986192	PD-L1	34	NSCLC	13 patients with NSCLC with [^89^Zr]Zr-Nivolumab and [^18^F]F-BMS
[^89^Zr]Zr-DFP-durvalumab	PD-L1	36	head and neck cancer	33 patients with recurrent or metastatic squamous cell carcinomas of head and neck
[^68^Ga]Ga-NOTA-RW102	PD-L1	37	preclinical in different PD-L1-expressing solid tumors and NSCLC patients	10 patients with NSCLC
[^68^Ga]Ga-NOTA-ABDRW102	PD-L1	37	preclinical, different PD-L1-expressing solid tumors	no patients
[^64^Cu]Cu-NOTA-ABDRW102	PD-L1	37	preclinical, different PD-L1-expressing solid tumors	no patients
[^89^Zr]Zr-DFO-ABDRW102	PD-L1	37	preclinical, different PD-L1-expressing solid tumors	no patients
[^68^Ga]Ga-NOTA-(hPD-L1) Nbs	PD-L1	38	preclinical, PD-L1-expressing tumors	no patients
[^18^F]F-NOTA-ZPD-L1_1	PD-L1	39	preclinical, PD-L1-pos. and -neg. tumors	no patients
[^89^Zr]Zr-BI 754111	LAG-3	40	head and neck cancer, NSCLC	2 patients with head and neck cancer, 4 patients with NSCLC
[^89^Zr]Zr-IAB22M2C	CD-8	41	melanomas, lung tumors, HCC	6 patients (1 melanoma, 4 lung cancer, 1 HCC)
[^89^Zr]Zr-DFO-CD4	CD-4	42	preclinical, breast cancer	no patients
[^89^Zr]Zr-DFO-CD8	CD-8	42	preclinical, breast cancer	no patients
[^89^Zr]Zr-trastuzumab	HER-2	43	breast cancer	5 patients with metastatic breast cancer
[^89^Zr]Zr-pertuzumab	HER-2	44	breast cancer	6 patients with HER-2-positive metastatic breast cancer
[^89^Zr]Zr-GSK2849330	HER-3	45	HER-3-positive solid tumors	6 patients with HER-3-positive solid tumors
[^89^Zr]Zr-cetuximab	HER-1	46	NSCLC, head and neck cancer	9 patients (6 with NSCLC and 3 with head and neck cancer)
[^89^Zr]Zr-lumretuzumab	HER-3	47	HER-3-positive solid tumors	20 patients with HER-3-expressing solid tumors
[^68^Ga]Ga-pentixafor	CXCR4	49–53	different solid tumors, hematologic malignancies	>800 patients with different solid and hematologic malignancies
[^89^Zr]Zr-bevacizumab	VEGF-A	54–60	breast cancer, renal cancer, NET, NSCLC	23 patients with breast cancer, 26 patients with renal cell carcinomas, 14 patients with NET, 7 patients with NSCLC
[^89^Zr]Zr-U36	CD-44	61	head and neck cancer	20 patients with head and neck tumors
[^89^Zr]Zr-rituxumab	CD-20	62	lymphomas	5 patients with CD20+ B-cell lymphoma
[^89^Zr]Zr-DFO-daratumumab	CD-38	63	multiple myeloma	10 patients with multiple myelomas
[^89^Zr]Zr-MMOT0530A	mesothelin	64	pancreatic cancer, ovarian cancer	11 patients (7 with pancreatic cancer and 4 with ovarian cancer)
[^68^Ga]Ga-NOTA-T4	TROP-2	66	solid tumors	10 patients with solid tumors

**Table 2:** A summary of non-FDG radiopharmaceuticals.

Tracer	Mechanism	Clinical	Diagnosis	Theragnostic
[^18^F]FLT	proliferation	yes	yes	no
[^18^F]FMAU	proliferation	yes	yes	no
[^11^C]methionine	amino acid transport	yes	yes	no
[^11^C]glutamate	amino acid transport	yes	yes	no
[^18^F]FET	amino acid transport	yes	yes	no
[^18^F]FDOPA	amino acid transport	yes	yes	no
[^11^C] and [^18^F]-labeled choline	lipid metabolism	yes	yes	no
[^11^C]acetate	lipid metabolism	yes	yes	no
[^18^F]FMISO	hypoxia	yes	yes	no
[^18^F]-FAZA	hypoxia	yes	yes	no
[^18^F]F-HX4	hypoxia	yes	yes	no
[^64^Cu]Cu-ATSM	hypoxia	yes	yes	no
[^68^Ga] and [^18^F]F-FAPI	tumor microenvironment	yes	yes	no
[^90^Y] and [^177^Lu]-FAPI	tumor microenvironment	yes	yes	yes
[^68^Ga]Ga-DOTA-TOC	SSTR 2	yes	yes	no
[^90^Y]-and [^177^Lu]-DOTA-TOC	SSTR 2	yes	yes	yes
[^68^Ga]Ga-PSMA-11	PSMA	yes	yes	no
[^18^F]-PSMA-1007	PSMA	yes	yes	no
[^177^Lu]-PSMA-617	PSMA	yes	yes	yes
[^68^Ga]Ga-pentixafor	CXCR4	yes	yes	no
[^90^Y]-pentixafor	CXCR4	yes	yes	yes
[^89^Zr]Zr-nivolumab	PD-1	yes	yes	no
[^89^Zr]Zr-atezolizumab	PD L-1	yes	yes	no
[^18^F]F-BMS-986192	PD L-1	yes	yes	no
[^89^Zr]Zr-DFP-durvalumab	PD L-1	yes	yes	no
[^68^Ga]Ga-NOTA-RW102	PD L-1	yes	yes	no
[^89^Zr]Zr-BI 754111	LAG-3	yes	yes	no
[^89^Zr]Zr-anti-CD8 minibody	T-cell	yes	yes	no
[^89^Zr]Zr-trastuzumab	HER	yes	yes	no
[^89^Zr]Zr-cetuximab	HER	yes	yes	no
[^18^F]FES	estrogen receptor	yes	yes	no
[^89^Zr]Zr-bevacizumab	VEGF A	yes	yes	no
[^89^Zr]Zr-U36	anti-CD44	yes	yes	no
[^89^Zr]Zr-rituxumab	anti-CD20	yes	yes	no
[^90^Y]Y-rituximab	anti-CD20	yes	yes	no
[^89^Zr]Zr-DFO-daratumumab	anti-CD38	yes	yes	no
[^89^Zr]Zr-MMOT0530A	mesothelin	yes	yes	no
[^68^Ga]Ga-NOTA-T4	Trop 2	yes	yes	no

## Data Availability

Not applicable.
